# Contribution of the Vertebral Posterior Elements in Anterior–Posterior DXA Spine Scans in Young Subjects

**DOI:** 10.1359/jbmr.090224

**Published:** 2009-02-16

**Authors:** David C Lee, Patricia P Campbell, Vicente Gilsanz, Tishya AL Wren

**Affiliations:** 1 Orthopaedic Center, Childrens HospitalLos Angeles, California, USA; 2 Department of Radiology, Childrens HospitalLos Angeles, California, USA; 3 Deparment of Biomedical Engineering, University of Southern CaliforniaLos Angeles, California, USA

**Keywords:** areal BMD, BMC, DXA, QCT, posterior elements

## Abstract

Because DXA is a projection technique, anterior–posterior (AP) measurements of the spine include the posterior elements and the vertebral body. This may be a disadvantage because the posterior elements likely contribute little to vertebral fracture resistance. This study used QCT to quantify the impact of the posterior elements in DXA AP spine measures. We examined 574 subjects (294 females and 280 males), age 6–25 yr, with DXA and QCT. QCT measures were calculated for the cancellous bone region and for the vertebral body including and excluding the posterior elements. DXA data were analyzed for the entire L_3_ vertebra and for a 10-mm slice corresponding to the QCT scan region. BMC and BMD were determined and compared using Pearson's correlation. The posterior elements accounted for 51.4 ± 4.2% of the total BMC, with a significant difference between males (49.9 ± 4.0%) and females (52.8 ± 3.9%, *p* < 0.001). This percentage increased with age in younger subjects of both sexes (*p* < 0.001) but was relatively consistent after age 17 for males and 16 for females (*p* > 0.10). DXA areal BMD and QCT volumetric BMD correlated strongly for the whole vertebra including the posterior elements (*R* = 0.83), with BMC measures showing a stronger relationship (*R* = 0.93). Relationships were weaker when excluding the posterior elements. We conclude that DXA BMC provides a measure of bone that is most consistent with QCT and that the contribution of the posterior elements is consistent in young subjects after sexual maturity.

## INTRODUCTION

DXA is the most commonly used method of assessing BMD in a clinical setting. Advantages of DXA include its low radiation, low cost, fast scan time, and precision.(1) However, because DXA is a projection technique, its accuracy is limited by the inability to quantify bone volume,(2) by inhomogeneity of the extraosseous tissues, and by inclusion of the cortical-rich, non–weight-bearing vertebral posterior elements in anterior–posterior (AP) spine scans.(3,4) These factors may be especially confounding in studies of growing children(5,6) and those undergoing dynamic changes in bone size and morphology.(7,8)

In DXA, X-rays pass through the body, and a cumulative attenuation is measured. Therefore, in the DXA bone region, the measured attenuation represents a combination of all soft tissue and bone in the path of the beams. The attenuation values are used to generate a 2D projection image and to calculate areal BMD (aBMD, g/cm^2^). Commercial DXA scanners also report the projected bone area and BMC (g).

QCT is an established and accurate alternative densitometry method. In contrast to DXA's projection technique, QCT data are reconstructed as 3D voxels represented by a linear attenuation coefficient, which can be converted into volumetric density. Furthermore, QCT images can be separated into different types of tissue, such as lean and adipose, as well as cortical and cancellous bone. Volumetric BMD (vBMD, mg/cm^3^) and BMC are conventionally measured in a cancellous region or the isolated vertebral body, because these are assumed to be the regions most strongly related to compressive fractures.

DXA bone measures are only moderately correlated with bone measures from QCT.(9,10) As a result, it is not uncommon for a subject to have conflicting bone measures or Z-scores from DXA and QCT.(9) Some disagreement between DXA and QCT outcomes might be expected because AP DXA measures include the posterior elements, whereas QCT measures generally exclude the posterior elements. Other differences in technique and the regions measured may also affect the accuracy and comparison of DXA and QCT vertebral measures, such as the assumption of a homogeneous extraosseous soft tissue region(11–13) in DXA measures and variations in marrow fat composition(14–16) in both measures.

The effect of the vertebral posterior elements, as well as aortic calcifications, can be eliminated using lateral DXA scans. However, this method is less commonly used because lateral projections of the spine may include the ribs, particularly in the upper vertebrae (T_1_–L_2_). In addition, lateral scans are still subject to DXA's other shortcomings, including traversing a larger amount of soft tissue in the medial–lateral direction. It is not clear whether the ability to avoid the posterior elements outweighs these limitations; studies comparing the efficacy of lateral and AP DXA have reported conflicting conclusions.(17–23) Currently, lateral DXA scans are used for monitoring patients with vertebral deformities(24) and detecting abdominal aortic calcifications(25) but not for clinical measurements of BMD.

The goal of this study is to use QCT to quantify the impact of the posterior elements in DXA AP spine scans. The amount of bone in the vertebra with and without the posterior elements will be evaluated with QCT, and the effects of different analysis regions will be assessed. We hypothesize that there will be good agreement between QCT and DXA bone measures when the modalities measure the same bone region (including the posterior elements) and that greater disparities will arise when typical QCT measures that exclude the posterior elements are considered.

## MATERIALS AND METHODS

### Clinical study

DXA and QCT scans of the lumbar vertebrae were performed in 574 subjects (294 females and 280 males), age 6–25 yr (mean, 15.5 ± 3.6 yr). All subjects were healthy, and prospective participants were excluded if they had any recent history of serious disorders or were taking medications that could affect bone, muscle, or growth. The Institutional Review Board for clinical investigations at Childrens Hospital Los Angeles approved the protocols for this study, and written informed consent was obtained from all parents and/or participants (for minors, parents provided consent and participants provided assent). The quantitative CT protocol was designed to keep radiation exposure to a level roughly equivalent to the exposure during a round-trip airplane flight across North America,(26,27) making its use in healthy subjects possible.

**FIG. 1:**
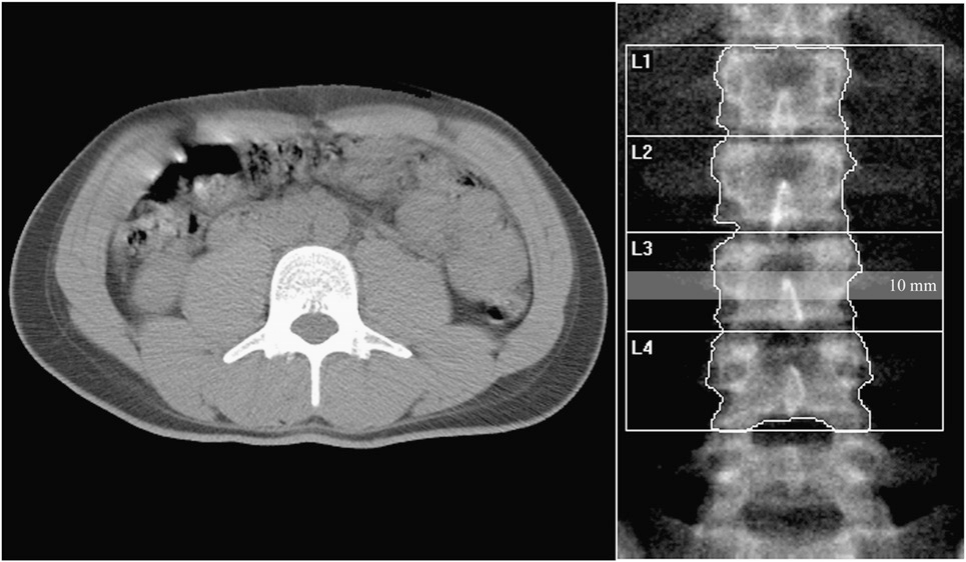
Sample images from the same subject with QCT (left) and DXA (right). The QCT scan location and thickness (10 mm) is shown in a shaded region on the L_3_ DXA scan.

For each subject, the DXA and QCT scans were done on the same day by a single radiology technologist. A DXA AP scan was performed on a Hologic Delphi W DXA scanner (Bedford, MA, USA) using the Fast Scan protocol (Fig. 1, right), and a transverse 10-mm cross-section through the midsection of L_3_ was obtained with QCT using a General Electric LightSpeed QC/i scanner (Waukesha, WI, USA) (Fig. 1, left). The specific techniques used have been described previously.(28,29)

### DXA analysis

The whole lumbar spine was scanned using four vertebral subregions (L_1_–L_4_). Two regions of interest (ROI) were defined for L_3_ using the Hologic QDR Software v11.2. The width of both ROIs was set at the default width of 116 lines (or 105 mm). The first ROI included the entire L_3_ vertebra. The second ROI adjusted the L_3_ subregion height to 10 mm centered about the midsection of the vertebra, which corresponds to the QCT slice (Fig. 1). Positioning of the ROIs was performed manually by the same technologist for all subjects. The Hologic software calculates aBMD, BMC, and projected area for each ROI (entire L_3_ and QCT region) according to standard DXA calculations.

### QCT analysis

**FIG. 2:**
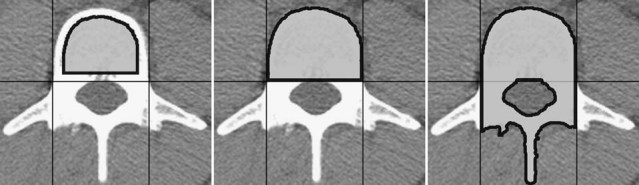
Regions of interest for the CT images. Left to right: cancellous bone region; isolated vertebral body; vertebral body including the posterior elements.

QCT data from L_3_ were analyzed using custom software developed in MATLAB 2006b (Mathworks, Natick, MA, USA). An algorithm was designed to automatically extract the vertebral shape and to separate the vertebral body from the posterior elements. Reported measures include vBMD and BMC of the cancellous bone region, the vertebral body excluding the posterior elements, and the vertebral body including the posterior elements (Fig. 2).

The vertebra is first identified by thresholding the image using the peak bone signal. Because there is high contrast between bone and the surrounding soft tissue, contours of the vertebra are easily extracted by edge detection. Efforts were taken to extract only the regions included in the DXA bone area, avoiding lateral aspects of the transverse processes. Thus, DXA's bone region was defined within two anterior–posterior lines through the lateral edges of the vertebral body, which are found by extending a tangent from the anterior edge of the vertebral foramen to the lateral edges of the vertebra. The same tangent line through the foramen serves to separate the vertebral body from the posterior elements, which include the spinous process, superior/inferior articular processes, and pedicels (Fig. 2).

The cross-sectional area (CSA) of each ROI was calculated by taking the integral of the region's contour. vBMD was determined by averaging the Hounsfield Units (HUs) contained in the CSA and converting HUs to hydroxyapatite equivalent density using a mineral phantom simultaneously imaged with the subject. BMC was derived by multiplying vBMD by the product of CSA and slice thickness (10 mm). The contribution of the posterior elements to bone mass was calculated by dividing the posterior element BMC by the total BMC (vertebral body plus posterior elements).

### Statistical analysis

Statistical analysis was performed using MATLAB 2006b (Mathworks). Pearson correlation coefficients were calculated to determine the relationship between the DXA and QCT measures, including both BMD and BMC, and between posterior element contribution and age. For the latter analysis, separate analyses were performed for mature and immature age groups. Because Tanner stage was not recorded for all subjects, subjects were assumed to be sexually mature at age 16 for females and age 17 for males. The Student's *t*-test was used to compare the posterior element contribution between females and males.

## RESULTS

**FIG. 3:**
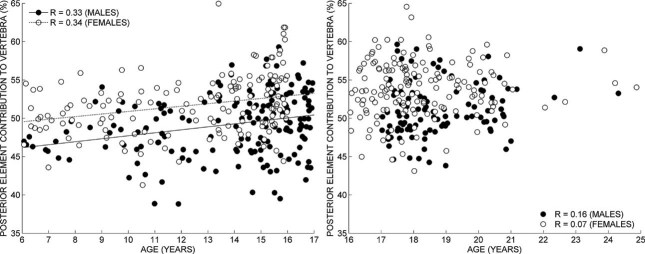
Posterior element contribution as a function of age.

The posterior elements accounted for 51.4 ± 4.2% (range, 38.8–65.0%) of the total bone content in the DXA scan region. There was a significant difference between males and females (males: 49.9 ± 4.0%, females: 52.8 ± 3.9%; *p* < 0.001). Additionally, the proportion of total BMC from the posterior elements increased with age for both younger males and females (*p* < 0.001) but did not change after age 17 in males and age 16 in females (*p* > 0.1; Fig. 3).

**FIG. 4:**
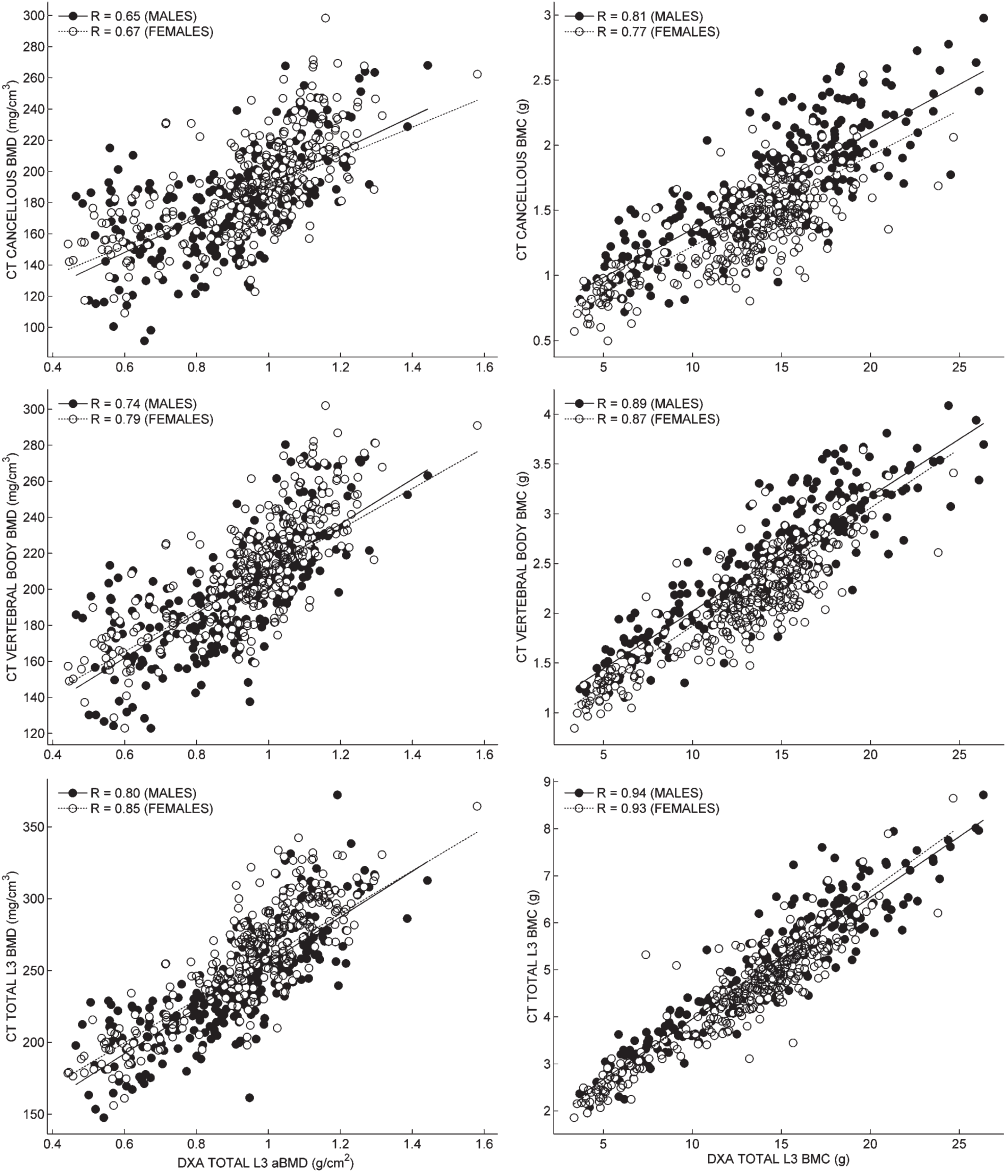
Comparisons of DXA and QCT measures.

Conventional measures of DXA aBMD (entire L_3_) and QCT vBMD (cancellous region) correlated only moderately (*R* = 0.66; Fig. 4; Table 1). This trend was the same in both males (*R* = 0.65) and females (*R* = 0.67). The correlation improved for QCT vBMD of the vertebral body, which includes the vertebral body's cortical shell (*R* = 0.77). The correlation improved further (*R* = 0.83) for QCT vBMD of the entire vertebra (including the posterior elements but not the transverse processes). Similar results were observed when the DXA scan region was adjusted to match the 10-mm QCT scan region, with comparable or slightly higher correlation coefficients.

**Table 1:** Correlations Between DXA/QCT BMD (Underlined) and BMC (Italicized)

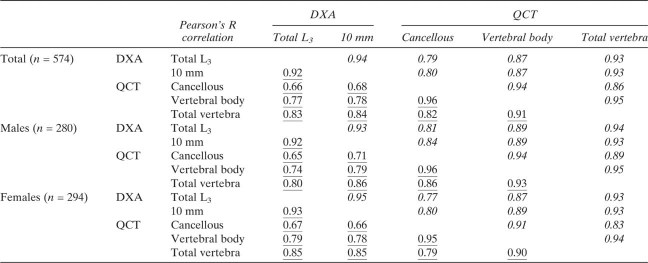

Higher correlation coefficients, but similar patterns, were observed when comparing BMC between DXA and QCT (Fig. 4). The correlation progressively increased as QCT measures moved from the cancellous region (*R* = 0.79) to the vertebral body (*R* = 0.87) to the entire vertebra (*R* = 0.93; Table 1). Correlations also increased or did not change significantly when the 10-mm DXA region was used.

## DISCUSSION

We found that the posterior elements contributed approximately one half of the total bone content in the vertebra. Others have found similar results in vitro using ash weight analysis.(30) Nottestad et al.(30) found that approximately one half of the mineral of L_3_ is in the vertebral body (43% for females and 51% for males) and that of the bone within the L_3_ vertebral body, trabecular bone accounts for less than one half (39% for females, 27% for males). The results in this report corroborate those previous studies in adult cadavers and extend their conclusions to children. Although females had a slightly higher proportion of bone mass in the posterior elements than males, the variability of the posterior element contribution was small in both sexes (SD ∼ 4%). In addition, the influence of the posterior elements stabilizes after puberty (Fig. 3). Therefore, adjustments to remove or avoid the posterior elements, such as with lateral DXA, may not be necessary for DXA bone measures in young, healthy subjects after puberty.

In contrast, caution should be exercised when interpreting DXA aBMD values in growing children because growth of the vertebrae is disproportional. Before age 16 for males and age 17 for females, the proportion of bone mass in the posterior elements seems to increase with age. This may contribute to increasing DXA aBMD values even though cancellous density in the vertebral body remains relatively constant in prepubertal children.(29)

Our results comparing DXA and QCT are consistent with other studies that found a moderate correlation between DXA aBMD and QCT vBMD.(9,10) Previous reports comparing DXA and QCT have acknowledged mismatched bone regions and other sources of error(9,10,28,31) as contributors to discrepancies between DXA and QCT measures but have not focused on the impact of the posterior elements in DXA measures. We found that much of the discrepancy between the two modalities is caused by the exclusion of the posterior elements in QCT analyses of the vertebra. When the posterior elements were included, we observed a stronger relationship between DXA and QCT measurements (vertebral body only: *R* = 0.77; vertebral body with posterior elements: *R* = 0.83).

Agreement between DXA and QCT bone measures was further improved by using the same unit of measure. Lack of the dimension along the path of the beam in DXA scans can cause size bias, an effect that reports different areal densities in bones of different sizes despite having the same volumetric density.(32) This error can be prominent in gender studies, because males tend to have larger vertebrae than females,(33,34) and in pediatric studies, because bone grows nonuniformly. Whereas it is possible to convert areal density to volumetric density through geometric or anthropometric scaling,(32) this may also introduce another source of error, particularly in children, whose bones grow disproportionately.

Instead, we converted both DXA aBMD and QCT vBMD to the same quantity, BMC (g), eliminating the confounding effect of comparing areal density with volumetric density and allowing for a clear understanding of the impact of the posterior elements. The correlation between DXA BMC and QCT BMC increased when the posterior elements were included (*R* = 0.87 to *R* = 0.93). This affirms the analysis based on density, which also showed a large increase in correlation with the inclusion of the posterior elements. The higher correlation between DXA and QCT using BMC corroborates other studies(10,35) that have suggested DXA BMC as a more reliable measure than DXA aBMD. We agree that BMC normalized for stature or body mass may be more informative than aBMD in evaluating skeletal status. Whole body BMC may also prove useful because it measures a much larger region. Ultimately, a comparison of bone measures in a prospective study of fracture risk is needed to identify the most clinically useful measures.

A limitation of this study is that the QCT measurement covered only a 10-mm section through the middle of L_3_. The vertebral posterior elements are highly irregular structures, and the morphology may be different in other vertebrae.(36) The impact of the posterior elements observed in the 10-mm midsection of L_3_ studied may not apply to the ends of L_3_ or to other posterior elements along the spine. Whereas it is possible to evaluate entire vertebrae with multislice QCT scans, the additional radiation exposure is not recommended.

In summary, the contribution of the posterior elements increases with age through the end of puberty but is relatively consistent in older adolescents and young adults. Therefore, the posterior elements have a negligible effect on DXA measures in these older subjects, but further study is needed to show the extent of their contribution in growing populations. Adding the vertebral posterior elements to QCT measures of the vertebral body resulted in a stronger relationship with DXA measures, especially for BMC. This supports DXA as a good measure of total bone in older adolescents and young adults.
